# The Other Side of Silence: A Psychiatrist's Memoir of Depression

**DOI:** 10.1192/pb.bp.115.053108

**Published:** 2017-04

**Authors:** Tom Brown

Linda Gask is an eminent academic psychiatrist with an outstanding international reputation. I state this upfront because it is none too obvious from this book – owing to her self-effacing style – and in my opinion, it is very relevant. I should also declare an interest as Linda and I were in the same year at Edinburgh University's medical school and I have heard small snippets of this story from her over the ensuing years.

In this excellent book Linda Gask shows what may be achieved despite living with a recurrent depressive illness; hope emerges even from her darkest moments and this work should encourage many. It is striking for its frankness and honesty – no small achievement given that she clearly must have known it would be read not only by colleagues, but by patients past and present, some of whom would have known little about her. She even mentions her failure to pass the MRCPsych exam at the first attempt and describes her – surprising to some! – experience of how sensitive and supportive a very senior academic colleague was at this time.

The book chronicles her life and career and the impact of her illness, including thoughtful reflections on its roots (in her early life). She teaches us about depression through the mirror of her own illness and that of her patients, and brings this to life through the use of clinical vignettes. She emphasises the importance of both biological and psychosocial factors in the origins of this illness and her description of treatments is both fair and accurate. Her accounts of her interactions with patients are particularly helpful and should be of value to any doctor, whether trainee or senior. I especially valued her comments on those whose failure to improve is ascribed to personality disorder, which is, alas, an all too common tactic of many psychiatrists.

This is an exceptional book and should be read by many, both doctors and patients. The high-profile endorsements on the cover are entirely deserved. Some years ago Linda wrote another excellent book entitled *A Short Introduction to Psychiatry*. I gave it to many medical students to read. I will give this to many more.

